# Novel RNA chaperone domain of RNA-binding protein La is regulated by AKT phosphorylation

**DOI:** 10.1093/nar/gku1309

**Published:** 2014-12-17

**Authors:** Julia Kuehnert, Gunhild Sommer, Avery W. Zierk, Alena Fedarovich, Alexander Brock, Dzmitry Fedarovich, Tilman Heise

**Affiliations:** Department of Biochemistry and Molecular Biology, Medical University of South Carolina, Charleston, SC 29425, USA

## Abstract

The cellular function of the cancer-associated RNA-binding protein La has been linked to translation of viral and cellular mRNAs. Recently, we have shown that the human La protein stimulates IRES-mediated translation of the cooperative oncogene CCND1 in cervical cancer cells. However, there is little known about the underlying molecular mechanism by which La stimulates CCND1 IRES-mediated translation, and we propose that its RNA chaperone activity is required. Herein, we show that La binds close to the CCND1 start codon and demonstrate that La's RNA chaperone activity can change the folding of its binding site. We map the RNA chaperone domain (RCD) within the C-terminal region of La in close proximity to a novel AKT phosphorylation site (T389). Phosphorylation at T389 by AKT-1 strongly impairs its RNA chaperone activity. Furthermore, we demonstrate that the RCD as well as T389 is required to stimulate CCND1 IRES-mediated translation in cells. In summary, we provide a model whereby a novel interplay between RNA-binding, RNA chaperoning and AKT phosphorylation of La protein regulates CCND1 IRES-mediated translation.

## INTRODUCTION

The La protein (LARP3) is a cancer-associated RNA-binding protein (1–6) initially identified as autoantigen in patients suffering from lupus erythematosus and Sjogren's syndrome (7,8). The La protein is implicated in many steps of the cellular and viral RNA metabolism including processing of RNA polymerase III transcripts, micro RNA processing and mRNA stabilization (9–19). The multifunctional RNA-binding protein shuttles between the nucleus and the cytoplasm (2,20–22). Several reports suggest that La is involved in translational regulation of viral and cellular RNAs with structure 5′ untranslated regions (5′-UTRs) (1–3,6,23–32). Some of those mRNAs contain an internal ribosome entry site (IRES) in their 5′-UTR allowing translational initiation when cap-dependent translation is impaired (33–35). However, the molecular mechanism by which La supports mRNA translation is still inexplicable.

Human La protein has three RNA-binding surfaces: the N-terminal located La motif (LAM), the RNA Recognition Motif 1 (RRM1) and the non-canonical RNA Recognition Motif 2 (RRM2) located in the C-terminal extension characteristic for mammalian La protein (36). The RNA-binding motifs have been characterized at the structural level (37–41). While the LAM and the RRM1 are important for interacting with RNA polymerase III transcripts containing a oligoU trailer for ‘3′-termini recognition’ (41–43), the RRM1 and RRM2 are thought to act in a cooperative manner for ‘internal recognition’ of RNA sequences derived from hepatitis B virus (44) and all three RNA-binding motifs interact synergistically with Hepatitis C virus (HCV) RNA (45,46). Hence, it is reasonable to speculate that La promotes mRNA translation by binding mRNAs via its RRM1 and RRM2.

In addition to its RNA-binding activity, an RNA chaperone activity has been reported for the La protein. Initial reports suggested the ability of La to melt DNA:RNA hybrid molecules in an ATP-dependent manner (47,48). More recent studies provide experimental evidence for La's RNA chaperone activity facilitating group I intron *cis*-splicing and strand-annealing/dissociation assays *in vitro*, bacteria and yeast (49,50), and strongly suggest RNA chaperone activity for the La protein and other La-related proteins (43,51). In general RNA chaperones are consider to be involved in many cellular processes due to their ability to bind RNA molecules trapped in non-functional structures and to assist the folding into a functional RNA structure (52–56). It is important to note that most mRNAs translational regulated by La are containing an IRES element known to intensively fold into secondary structures. This observation strongly supports our hypothesis that the La protein, which was the first known IRES-transacting factor (ITAFs), requires its RNA chaperone activity to promote structural changes favorable for translation initiation. The mapping of La's binding site near the start codon of the HCV IRES (24,46,57) and the preferential binding of La to start codons embedded in a strong Kozak sequence (58), supports the hypothesis that La may promote translational initiation by assisting correct start site usage (30). Nevertheless, so far no consensus binding sequence for La has been established supporting the notion that the binding site for the La protein might be comprised of sequence and structural characteristics.

The C-terminal region of the La protein contains the nuclear localization signal, a nucleolar localization signal, a nuclear retention signal and a well-characterized casein kinase 2 (CK2) phosphorylation site (36,59–63). In addition, the C-terminal region is intrinsically disordered (64), and holds stretches of basic amino acids, which may be involved in its RNA interactions (65). Interestingly, the C-terminal region seems to be important to promote IRES-mediated translation since a mutant missing the C-terminal region is not able to promote poliovirus IRES-mediated translation (30).

We have shown recently that the La protein is overexpressed in solid tumors, supports proliferation and mobility of cancer cells and established that La promotes IRES-mediated translation of cyclin D1 (CCND1) in HeLa cells (3,4). The recently identified CCND1 IRES requires its full CCND1 5′-UTR (209 nts) for maximal activity and is regulated by hnRNPA1 protein in a phosphorylation-dependent manner (66,67). Herein we use the CCND1 IRES as model to validate the mechanism whereby La facilitates translation of mRNAs, which harbor a structured 5′UTR. We are able to show that La binds in close proximity to the CCND1 start site, that La has RNA chaperone activity (RNA helix destabilization) that is able to change the structure of the CCND1 start site region, and that the RNA chaperone domain (RCD) is located in the C-terminal region of La. Furthermore, we demonstrate that the RNA chaperone activity and that the AKT (Protein Kinase B) phosphorylation site are required to stimulate CCND1-IRES-mediated translation in cells. In summary, we provide novel experimental evidence that the helix destabilization activity is important for La to promote IRES-mediated mRNA translation, and is regulated by AKT phosphorylation.

## MATERIALS AND METHODS

### For oligonucleotides and plasmid information please see supplementary materials

#### *In vitro* transcription

RNA probes, non-radioactive as well as radioactive [^32^P]-CTP (Cytidine triphosphate) labeled, were synthesized using the MEGAshortscript High Yield Transcription Kit (Ambion) according to the manufacturer's instructions. For the transcription of internally labeled RNAs, reactions were assembled in the following order: 2 μl T7 10x reaction buffer, 8 μl of 75 mM T7 ATP-GTP-UTP Mix (18.75 mM each), 1.5 mM cold CTP, 40 μCi [α-^32^P]-CTP, 100 nM of DNA template containing a T7 promoter, 2 μl T7 enzyme mix and nuclease-free water ad final volume of 20 μl. For DNA templates <75 nucleotides, 150 nM of template DNA was used for the *in vitro* transcription. The reactions were incubated for 3.5 h at 37°C and subsequently treated with 1 μl TURBO DNase for 15 min at 37°C. Non-radioactive RNA transcripts were synthesized by using a similar reaction, but using a mix of 100 mM T7 NTP Mix (ATP, GTP, UTP, CTP: 25 mM each) instead of unlabeled and [^32^P]-labeled CTP.

The bicistronic reporter plasmid (3) was used as template for T7 RNA polymerase mediated *in vitro* transcription of capped and polyadenylated mRNA according to manufacture instruction (Ambion, mMESSAGE mMACHINE^®^ T7 Ultra Kit).

The *in vitro* transcribed RNA was purified using a glass-filter based MEGAclear Kit from Ambion according to the manufacturer's instructions. The RNA yield was determined in a 1:10 dilution by diluting 3 μl of RNA to 27 μl 1x TE buffer (10 mM Tris/HCL pH 8.0, 1 mM Ethylenediaminetetraacetic acid(EDTA)). The absorbance at 260 nm was spectrophotometrically determined using a NanoDrop. The RNA concentration was calculated based on *A*_260_ of 1 equivalent to 40 ng RNA/μl: RNA concentration = *A*_260_ ×10 × 40 ng/μl, with 10 being the dilution factor.

#### Protein purification

For the purification of the His-tagged recombinant proteins human La wild-type (LaWT) and mutants (LaΔ1, LaΔ2, LaΔ4, LaΔRCD, LaT389A, LaT389E, La-Δlinker and LaRRM1 and 2) were expressed in *Escherichia coli* BL21, then purified using Ni-NTA spin columns following the manufacturer's instructions (Qiagen Ni-NTA Spin Handbook). Protein solutions as well as buffers were either kept on ice, or at 4°C, at all times and 1x complete protease inhibitor (Roche) were added freshly to all buffers. Spin columns were equilibrated with 600 μl lysis buffer (50 mM NaH2PO4, 10 mM imidazole, 300 mM NaCl, 1 mg/ml lysozyme, 1% (w/v) complete protease inhibitor) by centrifugation at 890 x *g* for 2 min prior to binding of His-tagged proteins to the Ni-NTA resin. For binding 600 μl of the cleared lysate containing the protein of interest were loaded on a spin column and centrifuged at 270 x *g* for 5 min. This step was repeated until all of the lysate was applied to the spin column. The flow through was collected and re-applied twice in order to ensure high efficient protein binding by saturating the Ni-NTA resin with His-tagged protein. The columns were subsequently washed six times with 600 μl wash buffer (50 mM NaH2PO4, 42.5 mM imidazole, 1 M NaCl, 0.11% (v/v) Triton X-100, 1% (w/v) complete protease inhibitor) for removal of contaminants, which were nonspecifically adsorbed by the resin, by centrifugation for 2 min at 700 x *g*. Proteins were eluted three times in 150 μl elution buffer (50 mM NaH2PO4, 300 mM imidazole, 300 mM NaCl, 1% (w/v) complete protease inhibitor) and centrifuged for 2 min at 700 x *g*. Proteins were dialyzed against dialysis buffer (10 mM Tris/HCl pH 7.4, 150 mM NaCl, 3 mM MgCl2, 0.5 mM EDTA, 5% glycerol). Protein concentration was determined and aliquots of the dialyzed protein were analysis by sodium dodecylsulphate-polyacrylamide gel electrophoresis (SDS-PAGE) and subsequent Coomassie staining.

#### Electrophoretic mobility shift assay

##### EMSA using radioactive labeled RNA

Several recombinant La mutants were tested for their ability to form complexes with the 5′-UTR of CCND1 mRNA. The binding affinity of recombinant La to the 5′-UTR was determined by titrating La against the RNA. A 6% native TBE (Tris-Borate-EDTA) polyacrylamide gel was prepared in a Mini-PROTEAN Tetra Handcast System, and was pre-run for 35 min at 240 V in 1x TBE buffer (45 mM Tris/HCl pH 8.5, 45 mM boric acid, 1 mM EDTA). The RNA was re-annealed for 10 min at 85°C in 1x RNA-binding buffer (20 mM Tris/HCl pH 8.0, 150 mM NaCl, 1.5 mM MgCl2, 0.5 mM EDTA, 0.5% (v/v) Nonidet P-40) and slowly cooled down to 37°C in a thermomixer. The binding reaction was prepared in a total volume of 20 μl in 1x RNA-binding buffer. Increasing concentrations of recombinant LaWT protein: 40, 80, 160, 320, 640 and 960 nM were placed in a 96-well microliter assay plate and 10 nM of re-annealed [^32^P]-labeled D1-FL RNA was added. A reaction with only the RNA transcript and no protein served as negative control. The samples were equilibrated for 10 min at room temperature before 2.5 μl RNA loading buffer (5% glycerol (v/v), 0.25% (w/v) bromphenol blue in 1x TBE buffer) was added and then samples were subjected to electrophoresis for 10 min at 240 V followed by 60 min at 100 V. The gel was removed from the glass plates, placed on Whatman filter paper, dried and exposed to a storage phosphor screen. The phosphor screen was scanned using a Storm PhosphorImager. The La–RNA complex (La–RNP) formation was quantified using the ImageQuant TL software and plotted as a function of the La concentration in Prism 5. The dissociation constant was determined using the one-site hyperbolic binding fit in Prism 5.

The binding affinity of wild-type (WT) recombinant La protein to the D1-AUG RNA oligoribonucleotide, was determined by native electrophoretic mobility shift assay (EMSA) as described above. Increasing concentrations of recombinant His-tagged La protein of 10, 60, 200, 400, 600, 800 nM, 1 M, 1.6 and 3 μM were used. The La–RNA complex (La–RNP) formation was quantified using the ImageQuant TL software. The dissociation constant (*K*_D_) was determined in Prism 5 after plotting the La–RNA complex formation as a function of the La protein concentration.

##### Competitive EMSA

EMSA were conducted as described above, but increasing amounts of cold competitor RNA was added and equilibrated for 10 min with 160 nM recombinant La protein before 10 nM radioactive labeled D1-FL or D1-AUG RNA was added. Cold competitors were added in excessive amounts of 100 (10×), 250 (25×) and 500 nM (50×) in order to challenge the binding. The specificity of the assay was tested by utilizing the cold transcripts of the radiolabeled D1-FL RNA or the unlabeled D1-AUG RNA oligoribonucleotide as competitor RNAs.

##### Super-shift assay

Incubating the protein of interest with a highly specific antibody leads to a much larger complex when bound to the RNA (super-shift). Three hundred nanogram of recombinant La protein was incubated for 1 h at 4°C with 5 μl of the mouse monoclonal anti-La SW5 antibody in 10 mM Tris/HCl pH 7.4 and 20 units RNasin (Promega) in a total reaction volume of 10 μl. A parallel reaction with 5 μl of the isotype control mouse IgG2α,κ antibody (Dako) was performed. The antibody pre-incubated La protein was subsequently used for EMSA as described.

#### Fluorescence polarization assay

An alternative quantitative method to monitor RNA–protein interactions is based on the alteration in tumbling properties of a fluorescent ligand upon binding to a larger molecule. The RNA of interest, D1-AUG, was synthesized and 5′-end labeled with the fluorophore 6-carboxyfluorescin (6-FAM) by Integrated DNA Technologies, Inc. The lyophilized RNA was resuspended in 1x siRNA (Dharmacon) buffer to a stock concentration of 100 μM. The 5′-FAM-labed D1-AUG RNA stock solution was diluted to 10 μM in fluorescence polarization (FP) assay buffer (20 mM Tris/HCl pH 8.0, 150 mM NaCl, 1.5 mM MgCl_2_, 0.5 mM EDTA, 0.5% (v/v) IGEPAL^®^ CA-630), heated for 10 min at 80°C and slowly cooled down to 37°C. 5 mg/ml (w/v) bovine gamma globulin was added to a final amount of 100 μg/ml. The 1.0 μM recombinant La protein stock solutions in FP assay buffer were prepared, and the La protein and its mutants were placed at increasing concentrations (1–300 nM) in black 384-well plates. Ten nanomolar of re-annealed RNA was added to the proteins and FP assay buffer was added to a total volume of 100 μl per reaction. Hundred microliter of the FP assay buffer was used as blank sample. To determine the background polarization, 10 nM free 5′-FAM-labed D1-AUG RNA in FP assay buffer was used as control. The POLARstar Omega plate reader (BMG Labtech) was utilized to measure FP after 5 min incubation at room temperature. The assay was done in quadruplicates for each protein concentration, to reduce the standard deviation. A 5x RNA–protein master mix of 500 μl was prepared and 100 μl was added per well. The background polarization was subtracted from the blank corrected data. La–RNA complex formation was determined as a change in millipolarization (ΔmP) and plotted as a function of the La concentration in Prism 5. The dissociation constant, *K*_D_, was determined by the one-site (hyperbola) non-linear fit algorithm in Prism 5.

#### RNA chaperone assay

All reagents, dilutions, proteins, the 96-well plate was kept on ice. RNA chaperone assays (RCA) were performed in a final volume of 40 μl in a black 96-well plate. The molecular beacon MB-D1-AUG was diluted to 50 nM in RCA buffer (2.5 mM Tris/HCl pH 8.0, 0.2 mM MgCl_2_, 19 mM NaCl, 0.13 mM EDTA, 0.01% IGEPAL) and heat up for 10 min at 80°C and subsequently place on ice. Recombinant La protein was added to the wells containing dialysis buffer (10 mM Tris/HCl pH 7.4, 150 mM NaCl, 3 mM MgCl_2_, 0.5 mM EDTA, 5% glycerol) to reach a final volume of 20 μl. Subsequently, 20 μl MB-D1-AUG (on ice) was added to three wells containing La or just 20 μl dialysis buffer (Blank). The 96-well plate was placed into the pre-heated (37°C) micro-plate reader and fluorescence light emission was measure at indicated time.

#### *In vitro* AKT kinase assay

Recombinant LaWT or LaT389A (600 ng of each) were incubated with recombinant AKT-1 (1.0 μg, Active Motif) in a 20 μl reaction mixture containing 20 mM,Tris/HCl, pH7.4, 50 mM KCl, 5 mM MgCl_2_, 0.5 mM DTT, 0.5 mM ATP and 10 μCi [γ^32^P]ATP (3000 Ci/mmol; Perkin Elmer, Waltham, MA, USA). After incubation for 3 h at 30°C reactions were stopped by adding SDS-loading buffer, boiled and resolved on a 12.5% SDS–PAGE. Gels were dried under vacuum and analyzed by autoradiography.

To prepare AKT-1 phosphorylated LaWT protein for the RCA, 1.2 μg recombinant La protein was phosphorylates in 20 μl volume over night at 30°C in reaction buffer described above containing 2.0 μg AKT-1. After incubation, 200 μl RCA buffer was added and the reaction was transferred into VWR centrifuge units, spun for 5 min at 14 000 x *g* at 4°C, washed three times with 200 μl RCA buffer and eluted according manufacturer's instructions. The volume was adjusted to 40 and 20 μl were analyzed in the RCD assay as described above.

#### CCND1 IRES luciferase reporter assay

HEK 293 cells were purchased from ATCC (American Type Culture Collection), expanded and aliquots were stored in liquid nitrogen. Cells were cultured at 37°C in a humidified 5% CO_2_ atmosphere in complete advanced dulbecco's modified eagle's medium containing 2 mM L-glutamine (Life Technology) plus 10% fetal bovine serum (Gibco). For transfection 4 × 10^5^ HEK 293 cells were plated per well of a 6-well plate. The next day cells were transfected with plasmid DNA encoding gfp-tagged LaWT, LaΔRCD, LaT389A using X-tremeGENE HP DNA Transfection Reagent (Roche) according to the manufactures instructions. Two days later cells were collected and replated into 96-well plates (4 × 10^4^ cells/well). The following morning cells were transfected with 150 ng of capped and polyadenylated bi-cistronic RNA with CCND1 IRES or without an IRES (empty) using the TransiT^®^ mRNA kit (Mirus). Seven hours later cells were harvested and luciferase expression was determined using the Dual-Luciferase Reporter Assay (Promega). Renilla and firefly luciferase activity is blotted as percent, in which renilla and firefly luciferase activity measured for the empty bicistronic RNA was set as 100%.

## RESULTS

### The La protein binds in close proximity to the CCND1 translational start site

**Figure 1.**
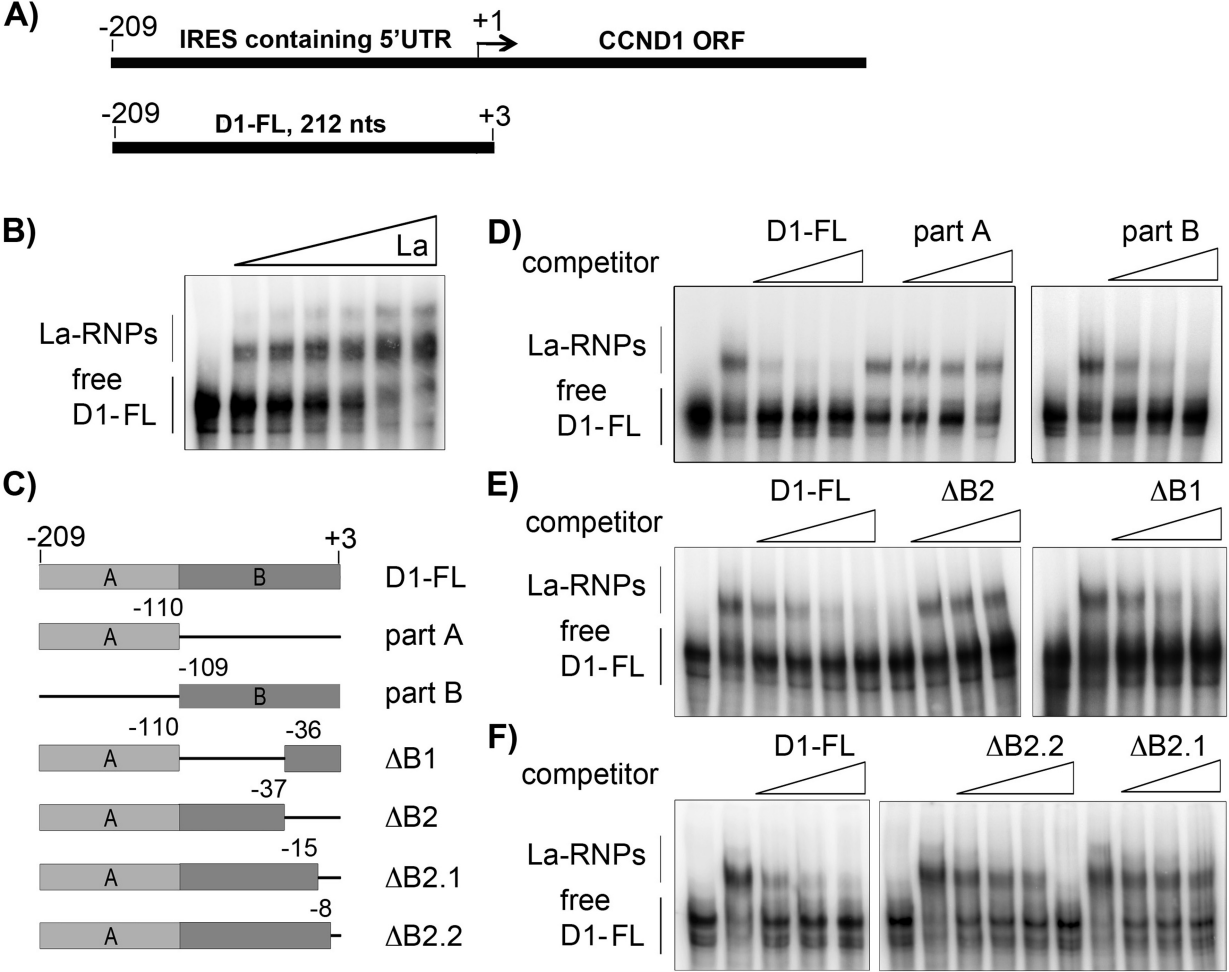
Mapping of La protein binding to CCND1 5′-UTR. (**A**) Scheme of the 5′-untranslated region (5′-UTR) of CCND1 (D1) containing an internal ribosomal entry site (IRES) and the full-length RNA probe (D1-FL) transcribed *in vitro.* (**B**) Electrophoretic mobility shift assays (EMSAs) demonstrating the binding of recombinant La protein to D1-FL RNA. Increasing concentration (40, 80, 160, 320, 640, 960 nM) of recombinant La protein were titrated to a constant concentration of 10 nM D1-FL RNA leading to the formation of La–ribonucleoprotein complex (La–RNP). (**C**) Scheme of *in vitro* transcribed competitor RNA spanning different regions of the CCND1 5′-UTR used in competitive EMSAs: part A = nts −209 to −110, part B = nts −110 to + 3, ΔB1 = internal deletion of nts −109 to −36, ΔB2 = nts −209 to −36, ΔB2.1 = nts −209 to −15, ΔB2.2 = nts 209 to −8. nts = nucleotides. (**D**), (**E**) and (**F**) competitive EMSA assays. The indicated unlabeled competitor *in vitro* transcripts where used at 10-, 25- and 50-fold excess over the labeled D1-FL RNA. Unlabeled D1-FL (D1-FL) RNA was used at 2.5-, 5-, 10-, 25- and 50-fold excess over labeled D1-FL RNA.

We have shown recently that the human La protein interacts with endogenous CCND1 mRNA and stimulates CCND1 IRES (D1-IRES)-mediated mRNA translation (3). Now we aim to explore the molecular mechanism by which La stimulates CCND1 mRNA translation. First we pursue to map the La-binding site within the 5′-UTR of the CCND1 mRNA. Therefore, we amplified the 5′-UTR including the start codon (D1-AUG, nts −209 to +3) of CCND1 mRNA by RT-PCR using a primer containing the T7-RNA polymerase promoter sequence. The DNA template was used to generate a ^32^P-UTP-labeled *in vitro* transcript referred to as D1-FL (Figure 1A, Supplementary Figure S1A). The D1-FL transcript was incubated with recombinant La protein and D1-FL:La complex formation was analyzed by EMSA. As shown (Figure 1B) increasing concentration of recombinant La (40, 80, 160, 320, 640, 960 nM) is shifting the D1-FL RNA by forming a major and a second, weaker La:RNA complex (La–RNP) of lower mobility. Quantification of EMSAs revealed a D1-UTR-FL RNA-binding affinity of about 41 nM (Supplementary Figure S1B).

To narrow down the La-binding site we generated two *in vitro* transcripts spanning the 5′-half of the 5′-UTR (part A (nts −209 to −110)) and the 3′-half of the 5′-UTR (part B (nts −109 to +3; Figure 1C). EMSA studies revealed that La protein binds preferentially to part B spanning nts −109 to +3 (data not shown). To further map the La-binding site we conducted competitive EMSAs using unlabeled transcript D1-FL as positive control and unlabeled transcript part A or part B RNA (Supplementary Figure S1C) to confirm that La binds preferentially to part B (Figure 1D). In addition, we generated a number of *in vitro* transcripts covering various regions of the 3′-terminal half of the CCND1 5′-UTR (Supplementary Figure S1D). Those unlabeled transcripts were used in competitive EMSAs to map the La-binding site (Figure 1E and F). Competition by transcript D1-FL served as positive control and competition by transcript ΔB1 (Δ nts: −109 to −36) suggested that La does not bind in the 5′-part of part B (Figure 1E). In contrast, transcripts ΔB2, ΔB2.1 or ΔB2.2 were not able to compete efficiently for La binding, suggesting that La favorably binds at the very end of the D1 5′-UTR, including the CCND1 start codon (Figure 1F).

**Figure 2.**
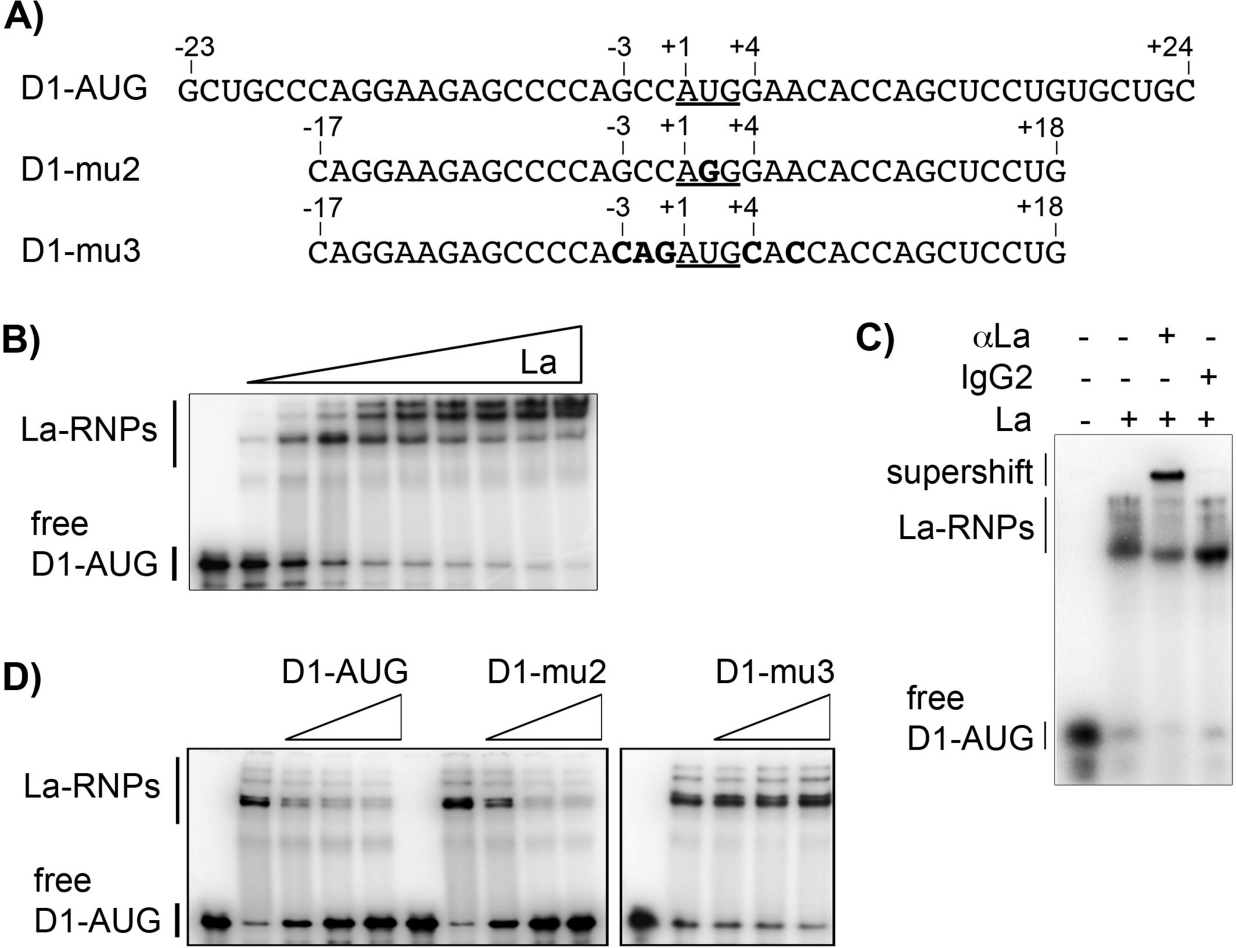
Binding of La protein requires a strong Kozak sequence. (**A**) Scheme of the synthetic RNA molecule D1-AUG spanning nucleotides −23 to +24 of the CCND1 mRNA. D1-mu2 represents a shorter version of D1-AUG with a single mutation in the translational start site. D1-mu3 represents a synthetic RNA molecule with mutation in the Kozak sequence surrounding the CCND1 start codon. (**B**) EMSA demonstrating binding of La to D1-AUG. Increasing concentration (10, 60, 200, 400, 800, 1600, 3000 nM) of recombinant La protein were titrated to a constant concentration of 10 nM D1-AUG RNA leading to the formation of La–ribonucleoprotein complex (La–RNP). (**C**) Supershift analysis demonstrating that La protein binds to D1-AUG RNA. αLa = anti-La antibody, IgG2 = control antibody. (**D**) The binding of La protein to D1-AUG RNA depends on a strong Kozak consensus sequence. Competitive EMSAs were performed to identify the role of the translational start site and its context. Competitive EMSA were performed using 10-, 50- and 100-fold excess amounts of unlabeled RNA and 10 nM [^32^P]-labeled CCND1-AUG RNA. As negative binding and competition control served a reaction without La protein and competitor RNA, respectively. Cold D1-AUG RNA and mu2 RNA, but not mu3 RNA are out-competing binding of La to radiolabeled D1-AUG RNA.

It has been shown recently that La binding engages the translational start site as well as surrounding nucleotides of the HCV's IRES (46,57). Furthermore, the preferential binding of La to RNA oligos with a start codon (AUG) embedded in a strong Kozak sequence has been demonstrated (58), suggesting that La may preferably recognize AUG-containing RNA elements. To test whether La binds preferentially in close proximity to the CCND1 start codon, we created a synthetic unlabeled or 5′-FAM labeled RNA molecule (D1-AUG, Figure 2A, Supplementary Figure S1F) spanning nts −23 to +24 (Figure 2A). We demonstrate by EMAS (Electrophoretic Mobility Shift Assay) (Figure 2B, Supplementary Figure S1F) and by FP assays (Supplementary Figure S1G) that La binds with an affinity of 80 and 76 nM, respectively, to D1-AUG-RNA. Next we used a La-specific antibody and a control IgG to demonstrate that the shift in D1-AUG-RNA mobility was caused by La protein. The La-specific antibody led to a super-shift (Figure 2C) demonstrating the formation of a specific La:D1-AUG-RNA complex.

It has been suggested that La contacts the HCV AUG (24,46,57). To test whether the D1-AUG is important for La binding as well, we designed an RNA oligonucleotide referred to as D1-RNA-mu2 (Figure 2A, Supplementary Figure S1E), in which we changed the U of the start codon to a G (AUG to AGG). In addition, to test whether a strong Kozak sequence context contributes to La binding, we designed the D1-RNA-mu3 RNA oligo, in which the Kozak sequence was changed from a strong CCND1 WT Kozak sequence (gccAUGg) to a weak Kozak sequence (cagAUGc, (Figure 2A, Supplementary Figure S1E). Competitive EMSAs using D1-RNA-mu2 and full-length recombinant La protein strongly suggested that the uridine of the D1-AUG is not critical for binding of La to the CCND1 start codon (Figure 2D). However, the Kozak sequence is definitively critical for binding of La to D1-AUG-RNA (Figure 2D). The analysis supports the notion that La binds to RNA sequences including and surrounding a translational start site, and that La prefers binding at start sites, in which the AUG is embedded in a strong Kozak sequence (58).

### The RRM1 and RRM2 of La are required and sufficient to bind D1-AUG-RNA

**Figure 3.**
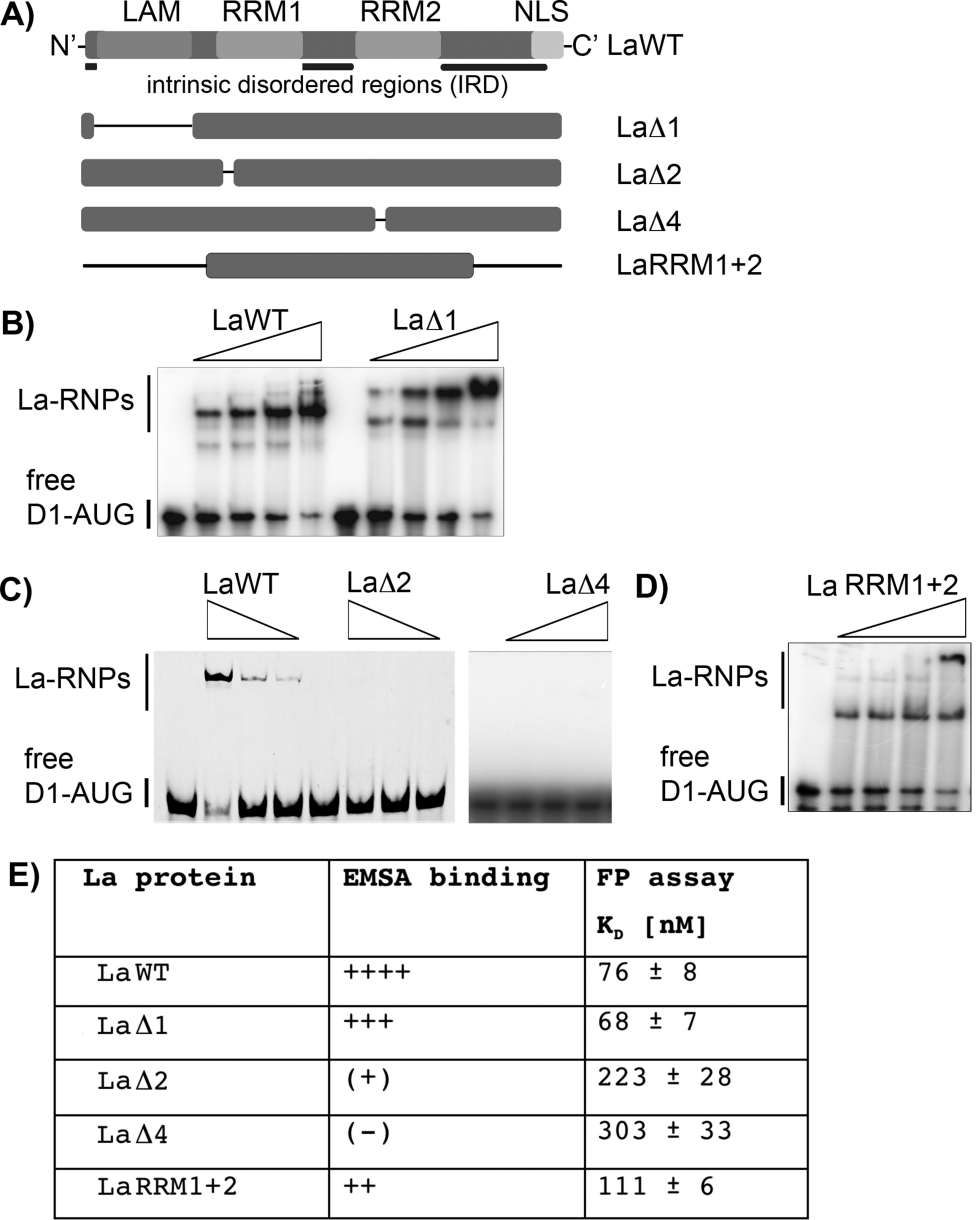
RRM1 and RRM2 are required for binding of D1-AUG RNA. (**A**) La protein mutants analyzed in RNA-binding studies. The scheme shows LaWT and its respective mutants; the black lines indicate the location of amino acid deletions. The LAM was deleted in LaΔ1, the RNP-2 consensus sequence was deleted in RRM1 and RRM2 for LaΔ2 and LaΔ4, respectively. The N-terminal region and the C-terminal region upstream and downstream of RRM1 and RRM2, respectively, are deleted in LaRRM1+2. For more details and purified proteins see Supplementary Figure S2. (**B**) (**C**) and (**D**) EMSAs were carried out with 40, 80, 160 and 320 nM recombinant La protein. Free D1-AUG RNA and La–RNPs are indicated. FP assays of all LaWT and mutant proteins were performed and are shown in Supplementary Figure S2. (**E**) Summary of D1-AUG RNA-binding studies using La protein mutants. The results of the RNA-binding studies by EMSA and FP show a high degree of similarity. The affinities determined by FP of LaΔ1, and LaRRM1+2 are similar to the *K*_D_ of the LaWT protein. This is also represented by the EMSA studies.

Next we aimed to define domains of the La protein required for binding of the CCND1-AUG-RNA. The La protein has three RNA-binding surfaces referred to as LAM, RRM1 and RRM2. Since our previous results suggested that internal RNA sequences/structures are bound via RRM1 and RRM2 of La protein (44), we propose that both RRM1 and RRM2 are required to bind the D1-AUG-RNA as well. To test this assumption we examined, by using EMSAs and FP assays, the ability of LaWT and several La mutants (Supplementary Figure S2A) to bind the ^32^P-labeled or a fluorescence-labeled D1-AUG-RNA (spanning nts: −23 to +24). La-WT and La-Δ1 (deletion of aa 11 to 99 (44)) bound D1-AUG-RNA with similar affinity (Figure 3A, B and E, Supplementary Figure S2B and D) suggesting that the LAM is not critical for binding. Analysis of La-Δ1 revealed two strong La–RNP complexes suggesting that the deletion of the LAM might allow the formation of multimeric complexes as observed earlier (44). In contrast, only low binding affinity was determined for La-Δ2 (deletion RNP2 sequence in RRM1, deletion of aa 113 to 119 (44)) or La-Δ4 (deletion of the RNP-2 sequence of RRM2, deletion of aa 235 to 242 (44)) (Figure 3A, C and E, Supplementary Figure S2A, C, E and G). Since the intact RRM1 could not compensate for a non-functional RRM2 and the functional RRM2 was unable to compensate for a non-functional RRM1, we conclude that both RRMs acting together to bind the D1-AUG-RNA demonstrate an interplay between different domains within La as suggested earlier (44). Next we generated La–RRM1 and 2 mutant containing only the RRM1 and RRM2 (Figure 3A) to test whether N- or C-terminal regions contributing to D1-AUG-RNA binding. As expected, La–RRM1 and 2 was able to bind D1-AUG-RNA (Figure 3D), however, the binding affinity was reduced (Figure 3E, Supplementary Figure S2F). This suggests that either the N- or C-terminal part of La contributes to its full binding affinity. The EMSA data and binding affinities are summarized in Figure 3E.

The findings suggest that the proposed intrinsic disordered linker region (64) between RRM1 and RRM2 provides flexibility required for RRM1 and RRM2 to contact the RNA. To test this notion we deleted the linker region (aa 170–230), and found that La-Δlinker was not able to bind D1-AUG RNA (Supplementary Figure S3). We conclude that both RRM1 and RRM2 are needed for binding of D1-AUG RNA, and that the intrinsic disordered linker region between both RNA recognition motifs is required for efficient recognition of the CCND1 translational start site region by the La protein.

### The RNA region surrounding CCND1 start codon can be restructured by La protein

**Figure 4.**
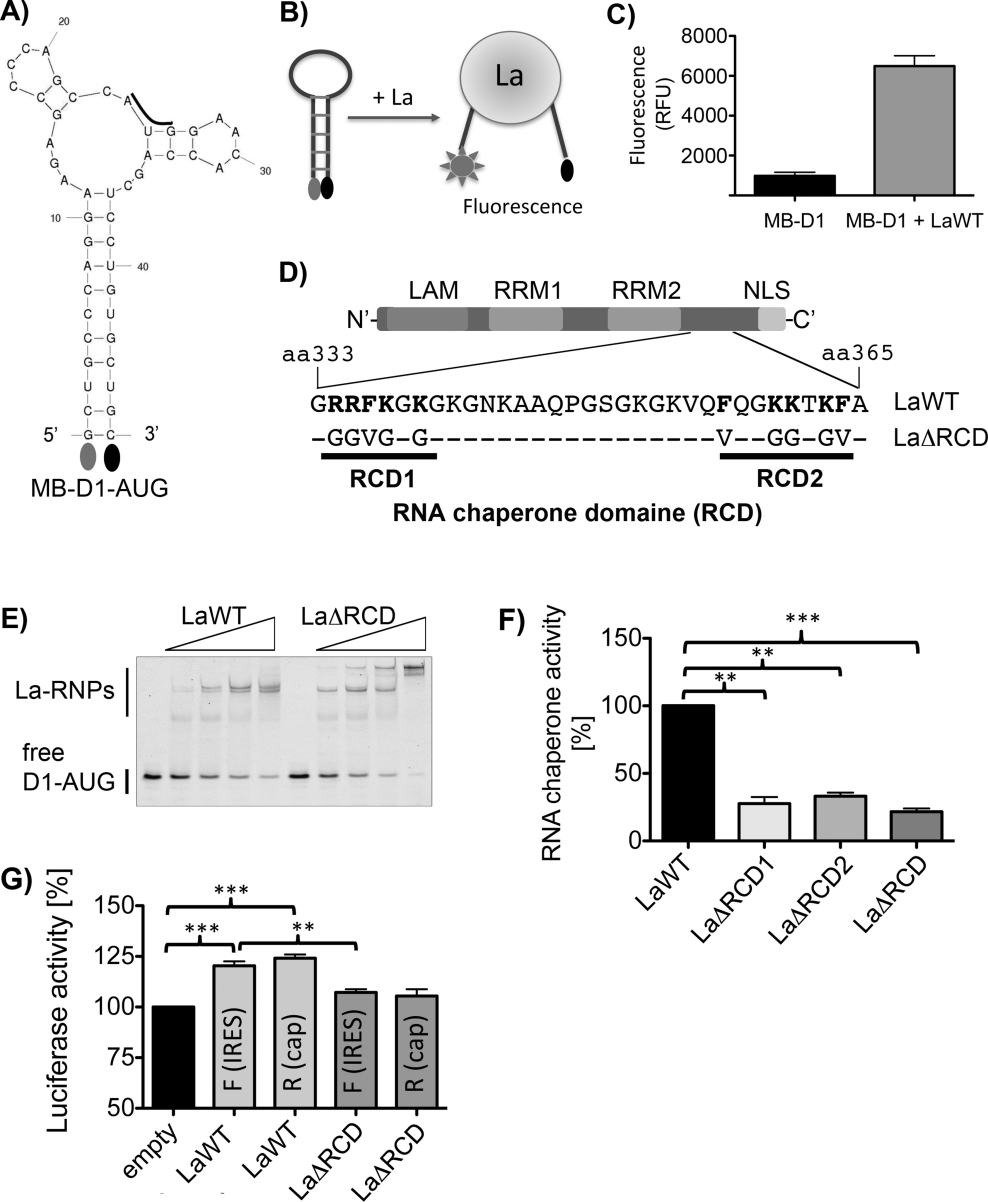
The RNA chaperone activity of La is required to stimulated CCND1 IRES-mediated mRNA translation. (**A**) Predicted structure of the D1-AUG molecular beacon (MB-D1-AUG): a fluorescein group was linked to the 5′-end and a quencher to its 3′-end of the D1-AUG RNA. (**B**) Scheme of the RCA used to measure helix-destabilization activity of recombinant La protein. (**C**) The La protein is destabilizing the helical region of molecular beacon MB-D1-AUG leading to a spatial separation of the fluorescein and quencher group. 25 nM of MB-D1-AUG was incubated with 300 ng of La protein and fluorescence emission was measured after 20 min at 37°C, n = 8. (**D**) Scheme of the C-terminal region of La containing the RCD. In mutant LaΔRCD amino acids indicated in bold were changed to amino acids shown below. (**E**) EMSA analysis demonstrating similar RNA-binding activity of LaWT and mutant LaΔRCD. Standard EMSA were performed as detailed in Materials and Methods and described in legends to Figures 1 and 2. (**F**) LaΔRCD1, LaΔRCD2, and LaΔRCD displayed strongly impaired RNA chaperone activity. 300 ng of recombinant LaWT or LaΔRCD mutants were incubated with 10 nM of D1-AUG-MB for 20 min at 37°C and fluorescence emission was recorded. Three independent protein preparations of LaWT and LaΔRCD mutants were analyzed in 3 or 4 independent experiments. RNA chaperone activity of LaWT was set as 100%. (**G**) LaWT but not LaΔRCD facilitated CCND1 IRES-mediated mRNA translation. Capped and polyadenylated bicistronic RNA without (empty) or with CCND1-IRES (IRES) was transfected into gfp-tagged LaWT or LaΔRCD expressing HEK 293 cells. Seven hours after transfection cap-dependent renilla (R) and CCND1-IRES-dependent firefly (F) luciferase activity was measured. Five independent experiments were analyzed (*n* = 5). Two-tailed *P*-value was determined by paired *t*-test (GraphPad). *P*-value < 0.01 (two asterisks) and <0.001 (three asterisks).

The structure of the HCV IRES revealed that the HCV start codon is located in a loop region of a bulged stem (68). Computer-based prediction (mfold (69)) of the folding of the CCND1 start codon region showed a single structure in which the D1-AUG is located in a looped region and in the formation of a long stem (Figure 4A). If we assume that the HCV-AUG and the D1-AUG are presented in a similar structural context, in which the AUG is present in a looped region closed by a stem of different length in living cells, we postulate that it would be of advantage to destabilize the AUG containing structural motif to facilitate the AUG recognition during translational initiation. To test whether La can act as RNA chaperone and is able to destabilize the predicted stem of D1-AUG RNA, we designed a molecular beacon (MB-D1-AUG RNA, Figure 4A). The 5′-end of the stem-closing base pair was labeled with fluorescein (5′ 6-FAM) and the 3’-end of the stem-closing base pair was labeled with a quencher (3′ Dabcyl). The principle of the RCA is shown (Figure 4B), and is based on the assumption that formation of the stem localizes the quencher in close proximity to the fluorescence group. In this conformation, the quencher would reduce light emission after excitation. We propose that binding of recombinant La to the MB-D1-AUG RNA will induce structural re-arrangements (helix-destabilization) causing a spatial separation of the fluorophore from the quencher. This is measured by an increase in fluorescence light emission. As shown, incubation of MB-D1-AUG RNA with recombinant La strongly increased the fluorescence emission, which demonstrates that La restructured the RNA theoretically by destabilizing the helical stem region (Figure 4C). Taken together we demonstrate that La can act as RNA chaperone and can destabilize the stem of the D1-AUG RNA.

### The RNA chaperone motif is located within the C-terminal region of La protein

We have demonstrated that La destabilizes the helical region of MB-D1-AUG RNA. Now we aim to identify the domain of La required for its RNA chaperone activity. Recently, it has been shown that the C-terminal intrinsic disordered regions (IDR) are important for La's ability to support the synthesis of various precursor RNA molecules (64), suggesting that the IDR harbors amino acid residues required for helix-destabilization activity. Earlier it has also been proposed that stretches of basic amino acids, located in the C-terminus of La, are engaged in RNA-binding (65). Here, we aim to demonstrate that those basic amino acid residues are involved in RNA-binding and/or helix destabilization and mutated two patches of basic amino acids and three phenylalanine residues located in the C-terminal region of La separately (LaΔRCD1, LaΔRCD2) or in combination (LaΔRCD, Figure 4D). We created the mutants by mutating the amino acids **RRFK**G**K** (aa 334 to 339) to **GGVG**G**G** (LaΔRCD1) and **F**QG**KK**T**KF** (aa 357 to 364) to **V**Q**GGG**T**GV** (LaΔRCD2, Figure 4D). Next recombinant LaΔRCD protein was purified (Supplementary Figure S4), and we tested whether those mutations affect the RNA-binding affinity of La to D1-AUG RNA. As shown LaΔRCD displays a similar ability to bind D1-AUG-RNA (Figure 4E) suggesting that the overall RNA-binding activity is not affected by those mutations. Analysis of LaΔRCD revealed two strong La–RNP complexes at high concentrations of La suggesting that the mutation introduced might allow the formation of multimeric complexes. Next, the recombinant LaΔRCD1, LaΔRCD2 and LaΔRCD proteins were analyzed for its chaperone activity by testing its ability to refold the molecular beacon MB-D1-AUG RNA. Compared to LaWT protein, LaΔRCD1, LaΔRCD2 as well as LaΔRCD mutant displayed a strongly impaired helix destabilization activity (Figure 4F). Taken together, we identified two small stretches of basic/aromatic amino acids located in the C-terminal domain contributing to the helix-destabilization activity. We refer to this region as the novel RCD of La.

### The RNA chaperone activity of La is required for IRES-mediated CCND1 mRNA translation in living cells

Above we have shown that the La protein binds in close proximity to the CCND1 start codon (CCND1-AUG), and that the RCD of La contributes to the helix-destabilization of D1-AUG RNA. Now we plan to demonstrate that La mutants with impaired RNA chaperone activity are defective in supporting CCND1 IRES-mediated translation in cell-based assays. To test this notion we generated capped and polyadenylated *in vitro* transcribed bicistronic CCND1 IRES reporter RNA, transfected the RNA into HEK 293 cells expressing gfp-tagged LaWT or LaΔRCD (Supplementary Figure S3C) and measured renilla and firefly luciferase activity after 7 h. Interestingly, these experiments demonstrate that LaWT not only stimulates CCND1 IRES-mediated firefly expression, but also cap-dependent renilla luciferase expression (Figure 4G). In contrast, both firefly and renilla luciferase expression was not stimulated by LaΔRCD. Because both renilla and firefly luciferase expression was elevated in LaWT expressing cells to similar levels we cannot describe IRES-mediated translation using the renilla:firefly luciferase ratio as often applied. This finding suggests that the RNA chaperone activity of La is required to stimulate both CCND1-IRES- and cap-dependent translation.

### Phosphorylation at threonine 389 by AKT-1 inhibits the RNA chaperone activity of La

**Figure 5.**
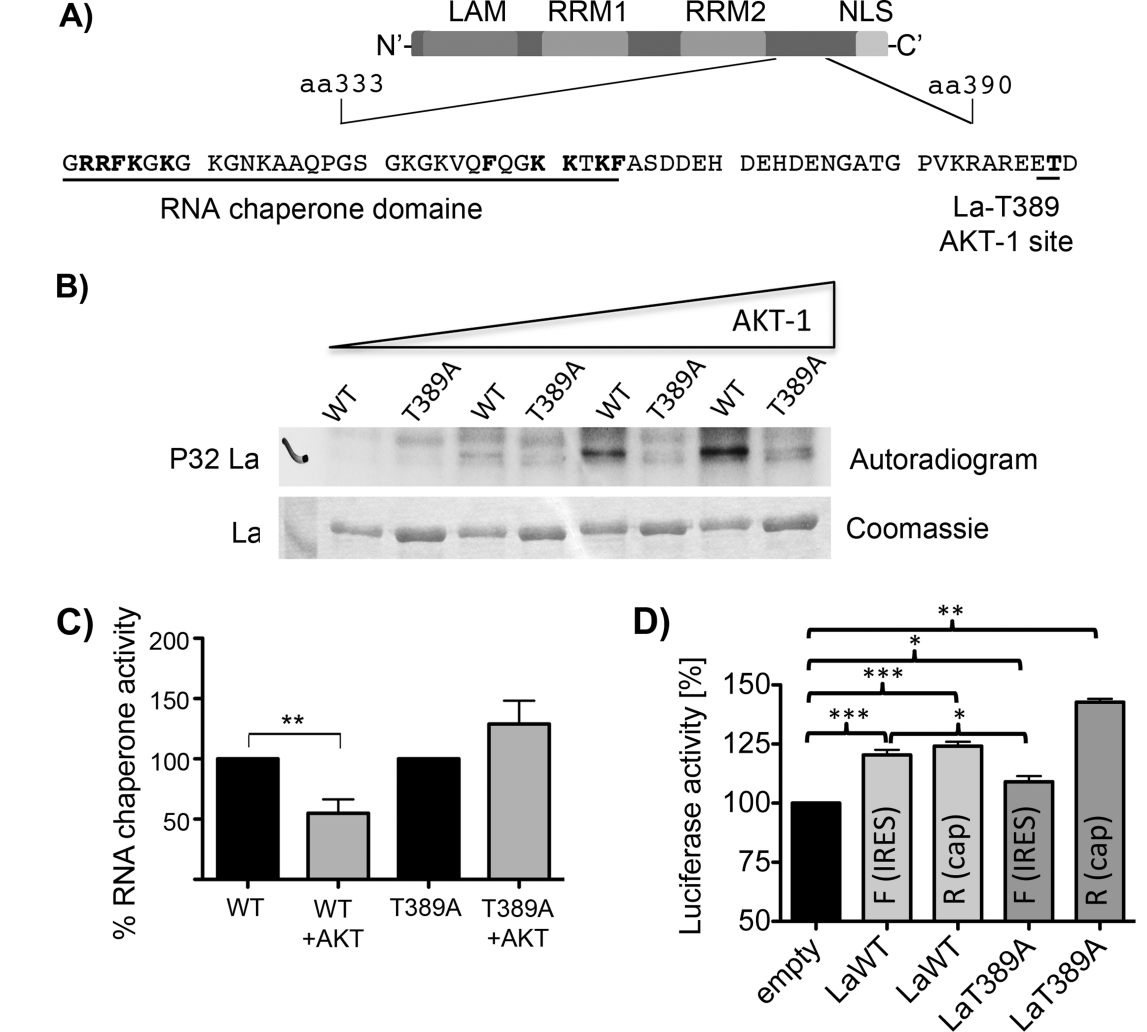
AKT phosphorylation regulates the RNA chaperone activity of La and stimulates CCND1 IRES-mediated translation. (**A**) Cartoon showing the RCD and the AKT phosphorylation site at threonine 389. The threonine 389 was changed to alanine (LaT389A) or to the phosphormimetic amino acid glutamic acid (LaT389E). (**B**) *In vitro* kinase assay demonstrated that AKT-1 phosphorylates La at T389. 600 ng of recombinant LaWT or mutant LaT389A was incubated in kinase buffer with increasing amount of recombinant AKT-1 in presence of γ^32^P-ATP and incubated for 2 h at 30°C. Protein phosphorylation was analyzed by SDS-PAGE and autoradiography of the dried gel. (**C**) AKT-1 phosphorylation reduces the RNA chaperone activity of recombinant La. 1200 ng of WT or T389A mutant La was phosphorylated by AKT-1 as described above and in ‘Material and Methods’ section. The buffer of the kinase and control reaction (no AKT-1) was changed to RCA buffer. *n* = 5, *P* = 0.0058. (**D**) LaT389A displays a strongly impaired ability to facilitated CCND1-IRES-mediated translation. Transfection of capped and polyadenylated bicistronic RNA without (empty) or with CCND1-IRES (IRES) into gfp-tagged LaWT or LaT389A. seven hours after transfection renilla (R) and CCND1-IRES-controlled firefly (F) luciferase activity was measured. Three independent experiments were analyzed (*n* = 3 or 4). Two-tailed *P*-value was determined by paired *t*-test (GraphPad). *P*-value < 0.05 (one asterisk), < 0.01 (two asterisks) and < 0.001 (three asterisks).

Recently, a genome wide study identified threonine 389 (T389) as AKT phosphorylation site located upstream of the newly identified RCD in La ((70), Figure 5A). To validate the AKT site, we changed threonine 389 to alanine (T389A), purified recombinant La proteins and performed an *in vitro* phosphorylation of LaWT and LaT389A by recombinant AKT-1. As shown, recombinant AKT-1 was able to phosphorylate LaWT, but not LaT389A (Figure 5B), which demonstrates *in* vitro that T389 is indeed an AKT-1 phosphorylation site. Interestingly, the phosphorylation site is located in the C-terminal domain of La in proximity to the RCD (Figure 5A). Hence, we aimed to test whether AKT phosphorylation regulates the RNA chaperone activity of La. Therefore, we phosphorylated LaWT or LaT389A with AKT-1 and incubated LaWT or LaT389A in the same buffer for the same time but without AKT-1 as control, changed the kinase buffer to RCA buffer using spin-filtration units and lastly performed RCA with native and phosphorylated LaWT. As shown the RNA chaperone activity of LaWT but not of LaT389A was strongly reduced by AKT-1 phosphorylation (Figure 5C). The dataset demonstrates that the RNA chaperone activity of La can be regulated by AKT-1 phosphorylation at T389 *in vitro*. Next, we tested whether mutation of T389 to alanine (LaT389A) or a phosphomimetic mutation (LaT389E) affects the helix destabilization activity of La. We purified three independent sets of recombinant La proteins (Supplementary Figure S5A) and measured their helix destabilization activity. We did not observed a significant difference in helix destabilization activity between LaWT, LaT389A or LaT389E (Supplementary Figure S5B), suggesting that we cannot replicate the results we observed by AKT-1 phosphorylation by mutating T389 to glutamic acid.

It has been shown earlier that AKT activity represses CCND1 IRES-mediated mRNA translation (66,67). To test whether AKT might regulate CCND1 IRES-mediated translation via T389 of La we transfected capped and polyadenylated bicistroninc RNA into gfp-tagged LaWT or LaT389A expressing HEK 293 cells and determined renilla and firefly luciferase activity as described above. Contrastingly, LaWT stimulated CCND1 IRES-mediated translation (firefly luciferase) as well as cap-dependent renilla luciferase expression but LaT389A was not able to promote CCND1 IRES-mediated translation (Figure 5D). However, LaT389A stimulated cap-dependent renilla luciferase expression (Figure 5D). The finding suggest that threonine 389 is important for La to stimulate CCND1 IRES-mediated translation, but not for cap-dependent translation. Note that previous literature suggests that the overexpression of La protein mutants can act in a trans-dominant negative manner (6,27,71) and therefore it appears reasonable to speculate that LaT389A inhibits endogenous La and thereby reduces CCND1 IRES-mediated mRNA translation.

In summary, we show that La can be phosphorylated *in vitro* by AKT-1 and that phosphorylation at T389 impairs the RNA chaperone activity of La protein. Furthermore, we demonstrate that overexpression of LaT389A mutant impairs specifically CCND1 IRES-mediated translation in cells.

## DISCUSSION

Herein we define for the RNA-binding protein, La, a novel RCD. We further demonstrate that the RNA chaperone activity of La is required to facilitate CCND1 IRES-mediated mRNA translation in cells and can be regulated by a nearby AKT-1 phosphorylation site (T389). These findings suggest a novel interplay between RNA-binding, RNA chaperoning and AKT-1 phosphorylation of the La protein and its regulation of CCND1 IRES-mediated mRNA translation.

Human La is suggested to facilitate (1–3,6,23,25–30,32), but also repress (26) IRES-mediated translation of various viral and cellular RNAs, however, the mechanism how La influences translation is still enigmatic. The findings that La (i) supports 48S complex formation during HCV and polio virus IRES-mediated translation (27,57); (ii) binds in close proximity to the HCV start codon (57); (iii) has the ability to correct start site selection (29,30) and (iv) binds preferentially to start codons embedded in a strong Kozak context (58), strongly suggest that La preferentially binds to selective start codons embedded in a specific sequence (Kozak) and structural context. Conclusively this information supports the following model: binding of La in close proximity to a start site leads—due to its RNA chaperone activity—to a destabilization of double-stranded RNA stretches surrounding the start site and easing start codon recognition by the scanning 43S complex to facilitate translation. To further support of this model, we show in this study that La binds in close proximity to the CCND1 start codon and that binding depends on a strong Kozak sequence (Figure 2). To our knowledge this is the second example of a La binding site in very close proximity to an authentic translational start site of an mRNA harboring an IRES in the 5′-UTR (46,57). Other La binding sites within the 5′-UTR of mRNAs are more upstream to the authentic translational start site (like BiP IRES: nt −207 to −115 (72); XIAP IRES: −64 to −34(28); MDM2 5′-UTR: −61 to −27(6)). In case of poliovirus IRES-mediated translation, the La protein binds to a region (nts 559–624) in the 5′-UTR of the polio virus mRNA in close proximity to two upstream open reading frames (uORF) upstream of the authentic translational start site and promotes the initiation at the authentic start site (29,30). Interestingly, MDM2 contains two regulatory uORFs (73), however, the La binding site in MDM2 5′-UTR was mapped downstream of those two uORFs (6). Therefore, many La-regulated 5′-UTRs are containing uORFs and more work is required to test the assumption that La contributes to authentic start site selection by regulating the usage of uORFs as previously suggested for poliovirus translation (29).

**Figure 6.**
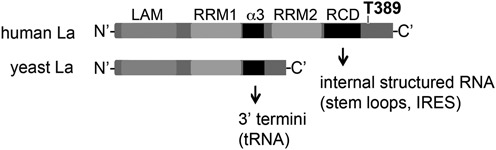
RCD of La proteins. Arrows indicate RCD located in yeast La (α3), mammalian La protein (RCD) and their potential RNA targets.

We propose that La facilitates mRNA translation not only by binding to the RNA, but also by changing the structure of the RNA. This view is supported by our data showing that La is able to bind and destabilize a helical RNA region in CCND1 mRNA surrounding the authentic start codon (Figure 4). This finding is of significance because it points to a potential function of La in translational initiation, namely, to remove structural obstructions. Recent literature showed that an element of human La (aa 1–235) containing the LAM, RRM1 and a C-terminal alpha-helix (α3), is required for strand-annealing, strand-dissociation as well as to rescue mutated tRNA presumably by assisting folding of misfolded pre-tRNAs ((50) Figure 6). Interestingly, the authors also showed that the C-terminal part of human La (aa 225–408) displayed RNA chaperone activity (50). In this context it is important to note that the newly defined RCD is required for helix destabilization and might be a specific feature of La proteins of higher eukaryotes harboring the C-terminal extension (Figure 6). Hence, it appears that distinct domains in La proteins are responsible for chaperoning of RNA targets containing a 3′-terminal oligoU trailer or internal RNA sequences within the 5′UTR, and that these domains may represent distinct features of lower and higher eukaryotic La proteins in pre-tRNA processing (43,50–51), translational control (this study) and miRNA metabolism (10,11). In this context it would be interesting to test whether expression of human LaΔRCD would affect pre-tRNA processing and/or the miRNA metabolism and whether yeast La is able to destabilize RNA structures as used in this study. Comparison of the C-terminal part of human, mouse, *drosophila* and zebrafish, La orthologs display strong similarities in respect to the presence of arginine, lysine and phenylalanine residues in the two regions (RCD1 and RCD2) that we mutated in the RCD (Supplementary Figure S6B). This suggests that these amino acids play an important role in the cellular function of La proteins in higher eukaryotes. It is also important to note, the RCD1 domain (aa 333 to 340) overlaps with the dimerization domain (aa 293 to 348, (71)) suggesting that mutations in this area impair dimerization, which may be a prerequisite for helix-destabilization. However, mutations introduced in RCD2 (aa 356 to 365, LaΔRCD2) are outside of the dimerization domain and are still reducing strongly the RNA chaperone activity of La (Figure 4F), suggesting that the defect in helix-destabilization activity of LaΔRCD is not due to impaired dimerization of La.

To define a functional importance of the RNA chaperone activity *in vivo*, we demonstrate that the same residues important for full RCD activity *in vitro* are required to stimulate CCND1 IRES-mediated mRNA translation in cell-based luciferase reporter assays (Figure 5), which supported our model that RNA chaperoning is required for La to stimulate IRES-mediated mRNA translation of CCND1. Interestingly, these studies also revealed that the RNA chaperone activity of La is required to stimulate cap-dependent translation of transfected bicistronic RNA, suggesting a more general role of La in protein synthesis. The stimulatory effect of LaWT on both cap- and IRES-dependent translation revealed that the renilla:firefly luciferase ratio, normally used to describe IRES-dependent translation, is not applicable with our bicistronic RNA transfection experiments. At this point we do not know why La stimulates cap-dependent translation of transfected RNA much more when compared to RNA transcribed from transfected DNA. We have to consider that the bicistronic RNA transcribed from transfected DNA will be exposed to nuclear proteins, will be spliced, has to be exported from the nucleus and therefore might be associated with a different set of proteins compared to RNA directly transfected into cells.

The finding that residues in the C-terminal region of La are required for RNA chaperone activity supports the notion that two distinct La domains are working together. Our data supports the view that RRM1 and RRM2 are required for initial binding of D1-AUG-MB and that the C-terminal RCD establishes a secondary contact to the RNA to assist helix-destabilization. This view would be in accordance with the ‘entropy model’ for RNA chaperones (55,74) proposing that RNA-binding proteins bind the RNA substrate. The interaction causes structural re-arrangements often in the disorder C-terminal domain of the RNA chaperone (52), allowing a second binding event to occur, which assists structural changes in the bound RNA molecule and—in our case helix-destabilization of D1-AUG-MB. Earlier it has been suggested that ITAFs contribute to unwinding RNA structures to support IRES-mediated translational initiation (33) and that the C-terminal part of La contains a region supporting poliovirus translation (30). Herein, we provide novel experimental data to support both earlier suggestions and findings. Interestingly, it had been recently shown that La is able to facilitate a *cis*-splicing reaction *in vitro* and was able to rescue splicing in bacteria cells (49). The authors propose that La has chaperone activity, which guides the folding of the RNA into a structure more favorable for splicing to occur (49). We used a molecular beacon approach to demonstrate that La possesses helix-destabilization activity, which may be important for cellular processes such as translational initiation and requires binding of La to internal RNA elements. However, more in-depth experimental methods are required to demonstrate distinct changes in RNA structure and to identify the contact sites between La protein and the target RNAs at nucleotide and amino acid levels.

The mutations we introduced in the RCD are mostly changes in amino acids with the potential to contact RNA, such as arginine, lysine and phenylalanine residues (75). Since mutating those residues within the C-terminal domain of La protein reduced the helix destabilization activity dramatically (Figure 4), we will aim in the future to demonstrate that those residues are engaged in RNA-binding as suggested in the model for RNA chaperones (52,74). We also observed a strong inhibition of La's helix-destabilization activity by AKT-1 phosphorylation (Figure 5C), suggesting that changing the hydroxyl group of T389 to a negative charged phosphate group has a detrimental effect on RNA restructuring. This data implies that the hydroxyl group might be involved in RNA-binding, as shown for other protein:RNA interactions (76) or for intra-molecular hydrogen bond formations stabilizing a La protein conformation important for RNA chaperoning. Efforts to show that LaT389A or LaT389E displays that impaired helix-destabilization activity failed, suggesting that only two negative charges at T389 impair helix-destabilization. Alternatively, the residues we identified to be important for helix-destabilization and the phosphorylation of T389 may play a role in the structural organization of the C-terminal disordered region upon RNA-binding. However, to make informed conclusions additional work is required to define the function of post-translational modification of the C-terminal disordered region in RNA-binding as well as to document structural rearrangements that occur upon RNA-binding (64) and AKT phosphorylation.

Recent reports demonstrate that CCND1 IRES is repressed in human cells with active AKT (66,67) and herein we show that the chaperone activity of La is impaired by AKT-phosphorylation, suggesting that AKT-mediate phosphorylation of La reduces CCND1 IRES-mediated translation. However, we observed a strong inhibition of CCND1 IRES-mediated translation in cells overexpressing LaT389A. LaT389A being a mutant which did not display any defect in RNA chaperone activity *in vitro*, thus suggests that the hydroxyl group of T389 is essential to support CCND1 IRES-mediated translation in cells by a mechanism not be recapitulated in *in vitro* RCA. Since we did not observed an increase in CCND1 IRES activity by expression of LaT389A, we assume that only a marginal fraction of La is AKT phosphorylated under the conditions we used to test the CCND1 IRES activity in cells. Other accusations may be that the hydroxyl group of T389 is required for CCND1 IRES-mediated translation in cells, or also that AKT phosphorylation of La actually stimulates CCND1 IRES-mediated translation in cells. The latter would be in agreement with recent work demonstrating that murine La shuttles in response to AKT activation to the cytoplasm and regulates mRNA translation (2,77), but would contradict the finding that the CCND1 IRES is repressed by active AKT in human cells (66,67). Interestingly, the C-terminal domain of La is also phosphorylated at S366 by CK2 (9), and it has been shown that overexpression of La S366A mutant reduces TOP mRNA translation in cells (62). Collectively our studies along with previous studies clearly show that post-translational modifications regulate the function of the La protein in mRNA translation, and more in depth studies are necessary to aid in understanding the molecular mechanism.

Because T389A and T389E La mutants displayed similar helix-destabilization activity as LaWT protein, we propose that the hydroxyl group at T389 mediates, yet unidentified, functions critical to promote CCND1 IRES-mediated translation. Mutation of the polar hydroxyl group to an aliphatic methyl group might affect the folding of the C-terminal domain, the interaction with other molecules such as RNA or other proteins due to the tendency of hydroxyl groups to form hydrogen bonds.

Summarizing the data shown herein suggest that the function of La in protein synthesis is not only mediated by its RNA-binding, but significantly, by its RNA chaperone activity, which requires the newly defined RCD located in the C-terminal intrinsic disordered region of La. In addition, we found that AKT-1 phosphorylation impairs the helix destabilization activity of La *in vitro* and that a threonine at position 389 (T389) is required for CCND1 IRES-mediated translation in cells. We conclude that the La protein is a modular protein requiring the concerted action of various domains to stimulate IRES-mediated mRNA translation, and that its function can be regulated by AKT signaling. We are convinced that this study provides novel information illuminating the function of the La protein during mRNA translation and its aberrant role in tumorigenesis.

## SUPPLEMENTARY DATA

Supplementary Data are available at NAR Online.

SUPPLEMENTARY DATA
