# Supplementary material for: Novel RNA chaperone domain of RNA-binding protein La is regulated by AKT phosphorylation
Source: Nucleic Acids Res. 2014 Dec 17;43(1):581–94. doi: 10.1093/nar/gku1309 (PMC4288197; doi:10.1093/nar/gku1309)
Supplement: SUPPLEMENTARY DATA [file supp_43_1_581__index.html]

Novel RNA chaperone domain of RNA-binding protein La is regulated by AKT phosphorylation — Novel RNA chaperone domain of RNA-binding protein La is regulated by AKT phosphorylation — SUPPLEMENTARY DATA 

# Novel RNA chaperone domain of RNA-binding protein La is regulated by AKT phosphorylation

## SUPPLEMENTARY DATA

**Files in this Data Supplement:**

- SUPPLEMENTARY DATA
