# Supplementary material for: Novel RNA chaperone domain of RNA-binding protein La is regulated by AKT phosphorylation
Source: Nucleic Acids Res. 2014 Dec 17;43(1):581–94. doi: 10.1093/nar/gku1309 (PMC4288197; doi:10.1093/nar/gku1309)
Supplement: SUPPLEMENTARY DATA [file supp_gku1309_nar-01941-r-2014-File008.pdf]

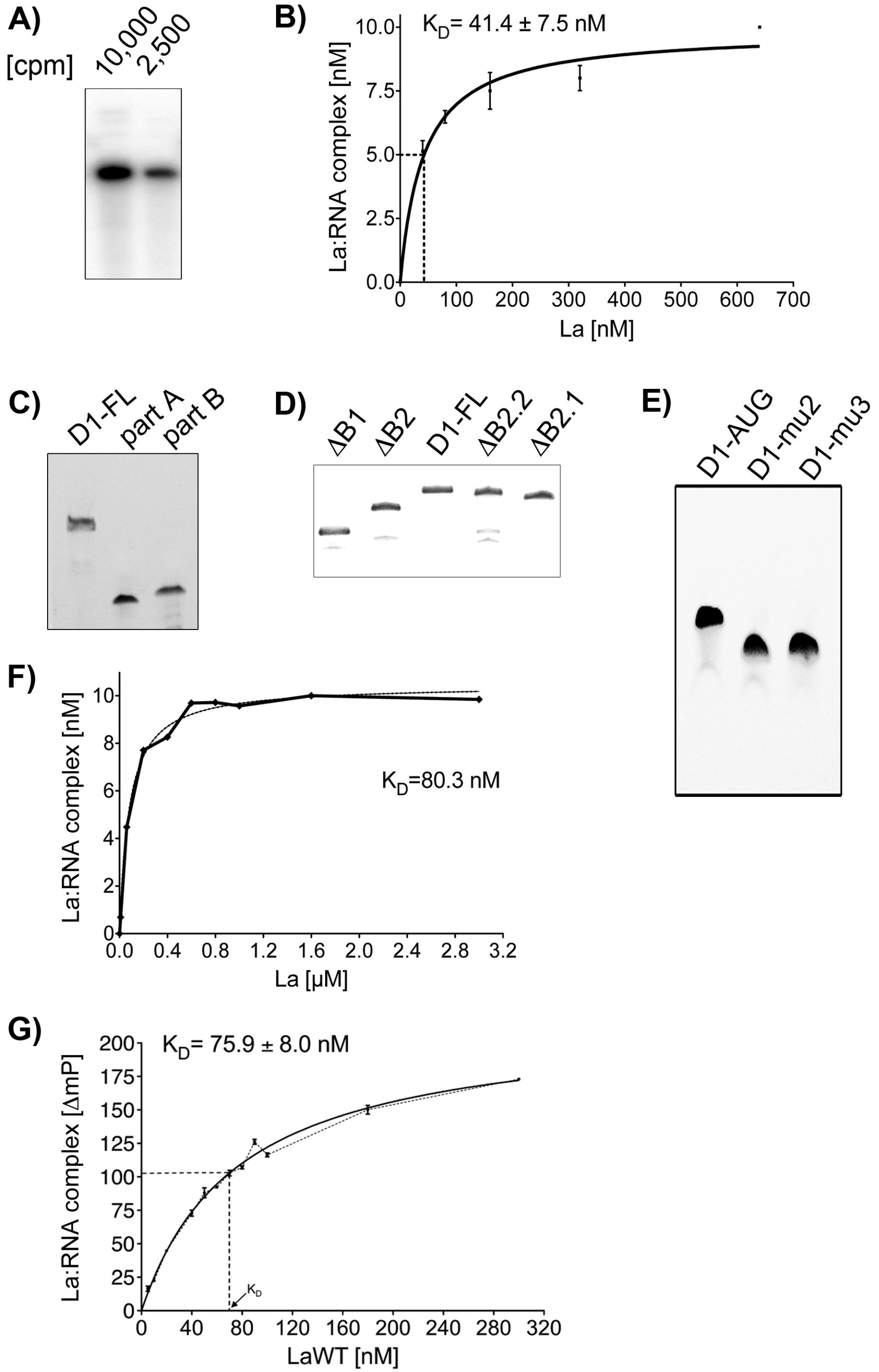

Kuehnert et al. Supp. Fig. 1

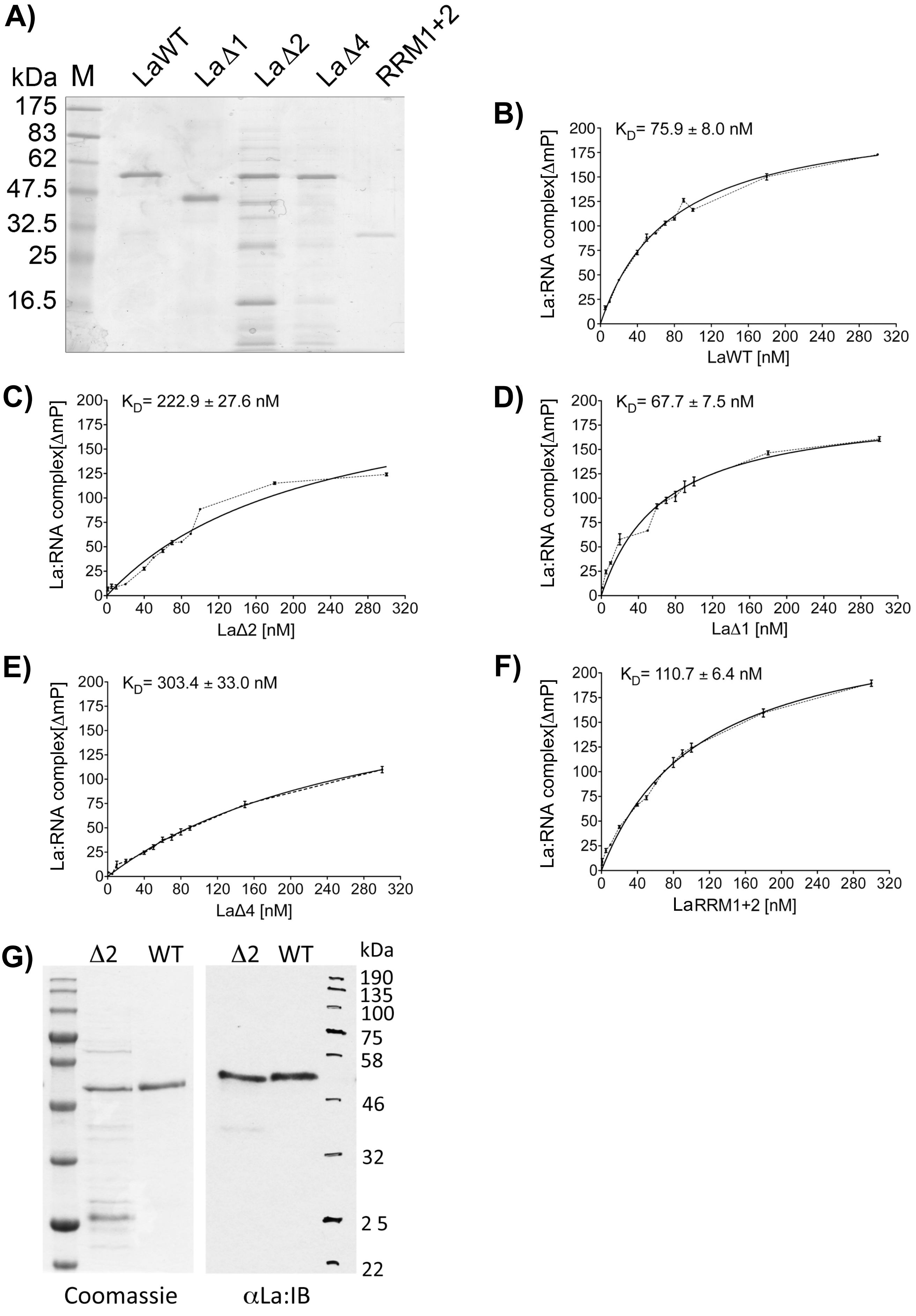

Kuehnert et al. Supp Fig. 2

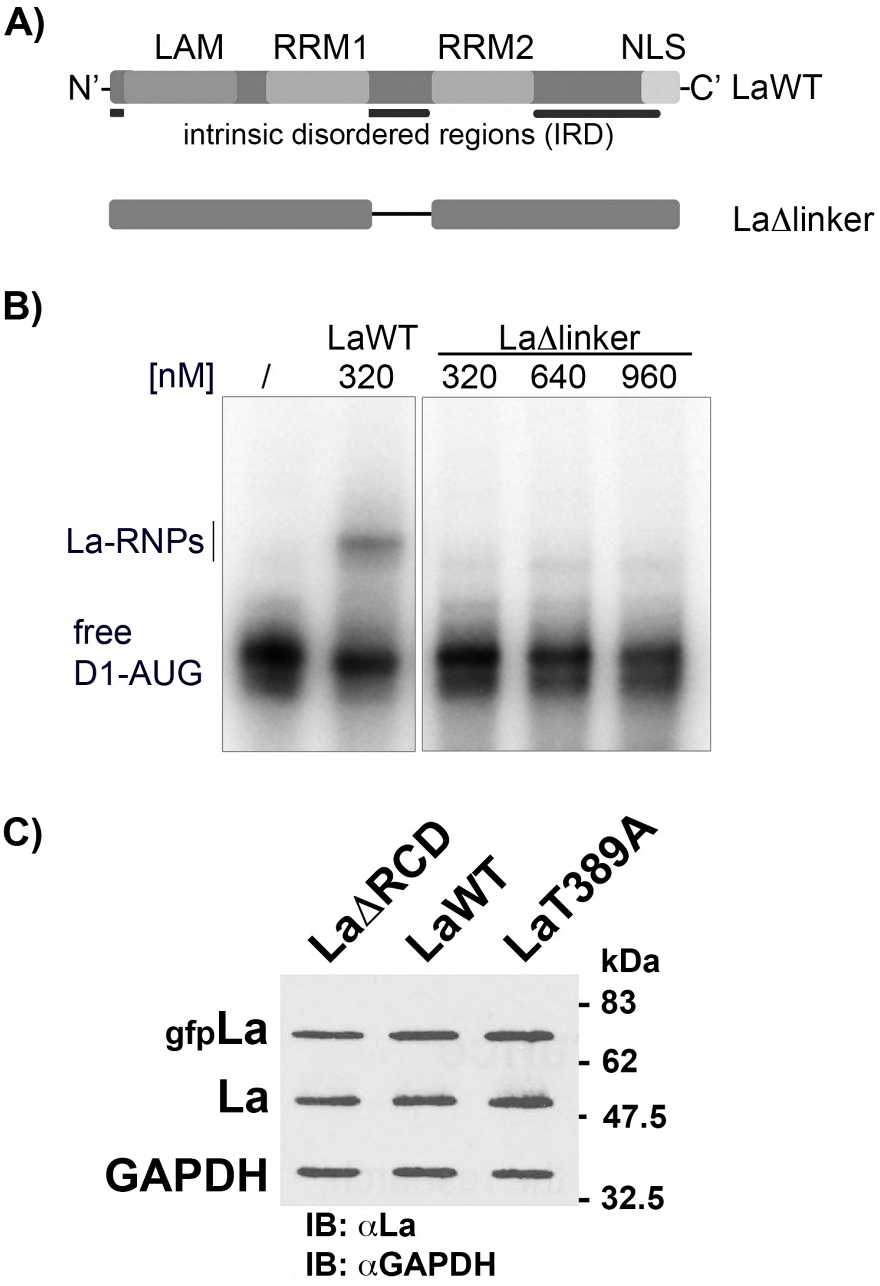

Kuehnert et al Supp Fig. 3

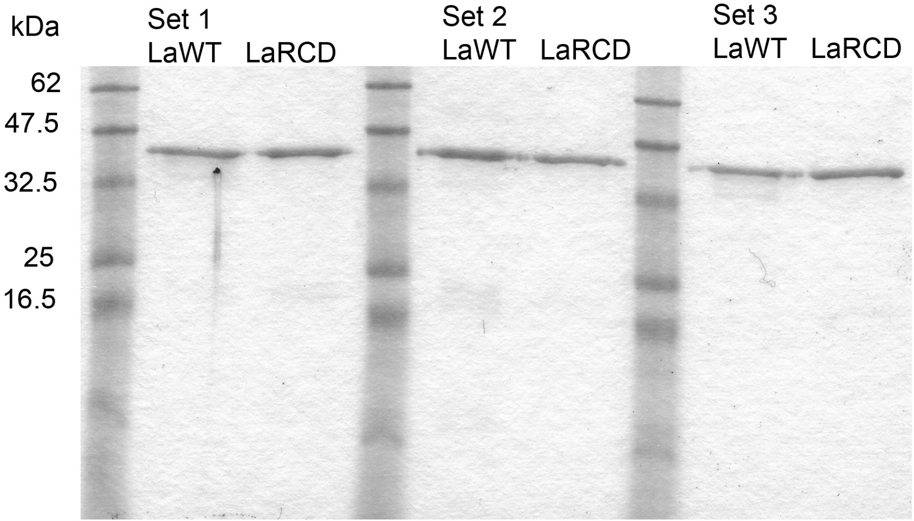

Kuehnert et al supp. Fig. 4

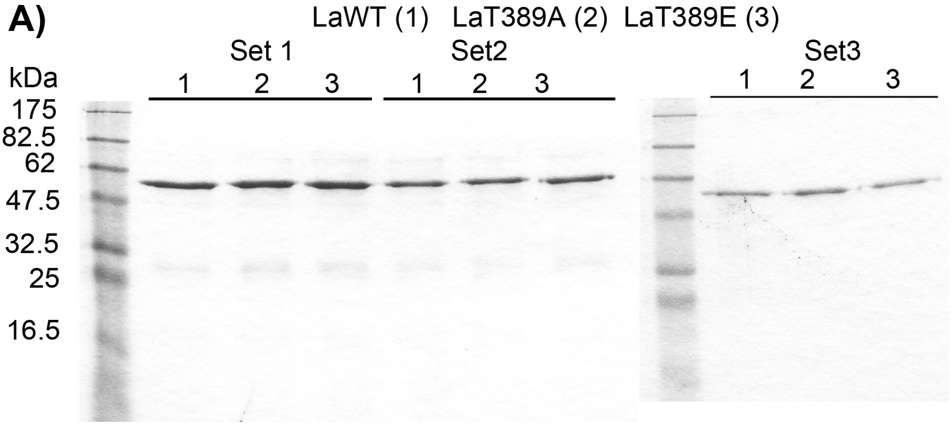

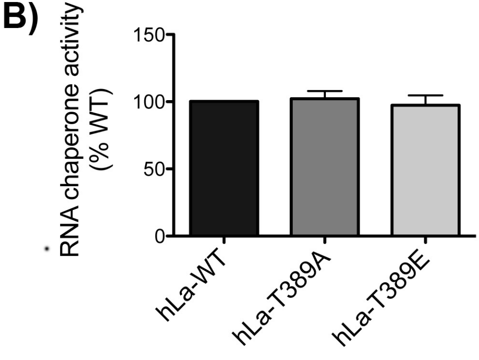

Kuehnert et al Supp. Fig. 5

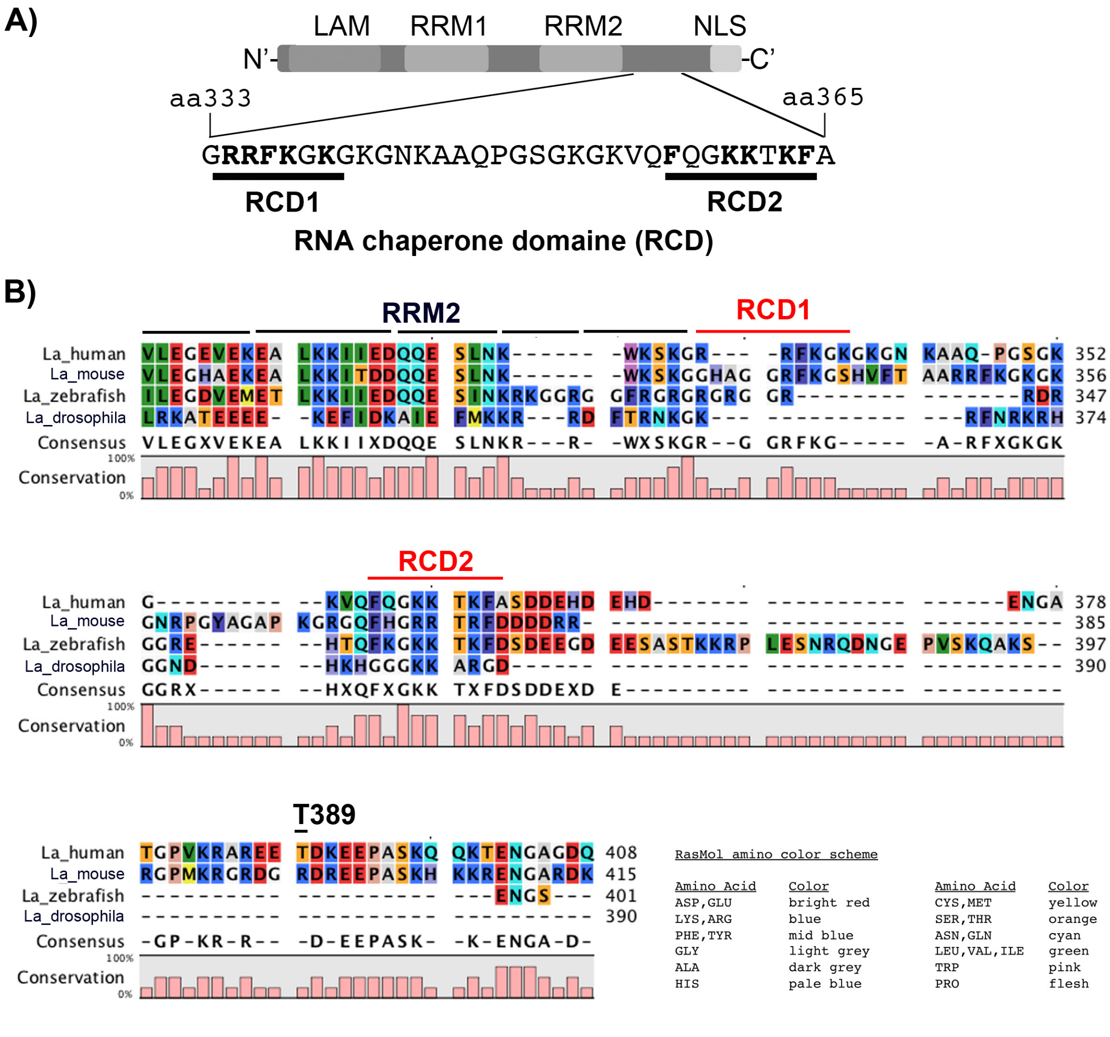

Kuehnert et al. Supp. Fig. 6

Supplemental Figures:

Supp. Fig. 1: Binding of recombinant La to the 5'-UTR of CCND1. A) The *in vitro* transcribed [^{32}P]-labeled D1-FL RNA was purified and radioisotope labeling assessed by scintillation counting. To assess the RNA integrity 2,500 and 10,000 cpm RNA were separated by denaturing PAGE and visualized using the Storm and ImageQuant TL phosphorimager system. The gel shows the high purity of the RNA represented by one RNA band for both concentrations. **B)** Recombinant La was analyzed for binding the D1-FL RNA by EMSA. The RNA concentration was held at 10 nM and 40, 80, 160, 320, 640, and 960 nM recombinant La were titrated for the EMSA assay. EMSA quantification of the bound RNA and plotted against the La concentration allowed the determination of the K_D of 45 ± 7 nM, D1 = CCND1, FL = full length. **C and D)** 400 ng of each unlabeled transcript was separated by denaturing PAGE and stained with ethidium bromide. **E)** Ethidium bromide staining of 400 ng cold D1-AUG, D1-mu2, and D1-mu3 RNAs separated by denaturing PAGE used in competitice EMSAs. **F)** Binding affinity of La:D1-AUG RNA interaction as determined by EMSA. 10 nM of labeled D1-AUG RNA was incubated with increasing amounts of recombinant La protein, 10, 60, 200, 400, 800, 1000, 1600, and 3000 nM and separated by a native EMSA. The La:RNA complex formation is plotted against the La protein concentration. The dissociation constant was determined by non-linear regression as 80.3 nM. **G)** Binding affinity for the La:D1-AUG RNA interaction as determined by fluorescence polarization assay. Human La was used in binding studies with 6-FAM-labeled D1-AUG RNA. The La:RNA complex formation as difference in polarization (ΔmP) was plotted against the La protein concentration. Using the one-site (hyperbola) non-linear regression fit the K_D was determined as 76 nM in Prism 5 from quadruplicates of two independent experiments (n=2).

Supp. Fig. 2: Binding affinity for the La mutants:D1-AUG RNA interaction as determined by fluorescence polarization (FP) assays. **A)** Equal molarities of the recombinant proteins, LaWT (47 kDa), LaΔ1 (36 kDa), LaΔ2 (46 kDa), LaΔ4 (46 kDa), LaRRM1+2 (25 kDa) were separated by SDS-PAGE and subsequently stained with Coomassie. The protein marker and the corresponding molecular weights in kDa are on the left. **B)** to **F)** Binding curves of LaWT and RNA-binding domain mutants LaΔ1, LaΔ2, LaΔ4, LaRRM1+2 as determined by FP assays using 6-FAM labeled D1-AUG RNA. The La:RNA complex formation as difference in polarization (ΔmP) was plotted against the La protein concentration. The dissociation constants were calculated using the one-site (hyperbola) non-linear regression fit in the Prism 5 software from quadruplicates of 2 independent experiments (n=2). **G)** Comparison of recombinant LaWT and LaΔ2 by Coomassie staining and La-specific Western blot analysis to demonstrate similar amount of full-length proteins. The two protein preparations were used in EMSAs shown in Fig. 3C.

Supp. Fig. 3: The linker between RRM1 and RRM2 within the La protein is required for RNA binding. A) Cartoon of LaWT and LaΔlinker mutant. B) EMSA analysis of binding of recombinant LaWT and LaΔlinker to D1-AUG RNA. C) La-specific Western blot analysis of HEK 293 cells transfected with gfp-tagged LaWT, LaΔRCD, or LaT389A. GAPDH was used as a loading control.

Supp. Fig. 4): Three preparations of recombinant LaWT and LaΔRCD proteins used in this study. 1 μg of recombinant proteins were separated on a 12.5% SDS-PAGE and stained by Coomassie.

Supp. Fig. 5: A) Three preparations of recombinant LaWT, LaT389A, and LaT389E proteins used in this study. 1 μg of recombinant proteins were separated on a 12.5% SDS-PAGE and stained by Coomassie. **B)** No significant difference in RNA chaperone activity was found comparing LaWT, LaT389A, and LaT389E proteins. Three independent preparations of LaWT, LaT389A, and LaT389E proteins were tested. 300 ng of recombinant protein was incubated with 10 nM of D1-AUG-MB for 20 min at 37°C and fluorescence emission was recorded. n=8.

Supp. Fig. 6: A) Scheme of the RNA chaperone domain of La. RCD1 and RCD2 represent regions of the RCD which were analyzed by designated mutations (LaΔRCD) in this study. **B)** Alignment of human, mouse, zebrafish and drosophila La protein sequences demonstrates sequence homology in RCD1 and RCD2. Regions corresponding to the human RCD1 and RCD2 amino acids are indicated. Threonine 389 is indicated and is only present in human La protein. The color scheme for the amino acids is shown.
